# Miscellaneous

**Published:** 1866-04

**Authors:** 


### MISCELLANEOUS.

### *The Metropolitan Health Bill of New York.*

The earnest desire of the medical profession of this city, and of the community at large, has at length been gratified by the actual passage of a bill for the protection of the health and life of the community. The urgent want for such provisions as are now embodied in the Metropolitan Health Bill has so long been felt, and so many measures have been taken by one party and another to effect the grand object, and the failures in their accomplishment have been so numerous, that it is really a matter of surprise, even to those who were reasonably sanguine in the matter, that we now have a law which, by the comprehensiveness of its scope and the liberality of its design, is well calculated to meet all the requirements of the case. It is true that the present bill is nothing more than the modification of the original one; but, after all, the alterations which have been made can have no direct tendency, as far as we can see, in interfering with its usefulness. The length of the bill forbids us giving our readers more than a mere synopsis of its provisions.

It creates a Metropolitan Health District, which has the same boundaries as the Metropolitan Police District. This district is in charge of a Board of Health, consisting of four Police Commissioners, the Health Officer of the port, and four Sanitary Commissioners—three of whom must be physicians, and one a resident of Brooklyn. The four Sanitary Commissioners shall hold office "respectively for the terms following—namely, one for one year, one for two years, one for three years, and one for four years; and until their successors shall be appointed and qualified." The term of office of each of the Commissioners, "after the expiration of the terms aforesaid, shall be four years."

The Board is to elect its own officers, and its President shall have all the power and authority given to the "City Inspector, in the six hundred and forty-sixth chapter of the laws of 1865, (passed May 1, 1865,) in respect to the making, awarding, or executing of a contract or contracts for street-cleaning, or any matter thereto pertaining."

The Sanitary Commissioners are to receive a salary of two thousand five hundred dollars per annum, and the Health Officer and Police Commissioners five hundred dollars per annum, as members of the Board; and all are forbidden to accept any other political or municipal office, or any nomination for such office, during their terms. Any member may be removed by the Governor, under the law relating to the removal of sheriffs.

The Board of Health has authority to appoint a Sanitary Superintendent, who must be an "experienced and skilful physician," and who shall, under its direction, "exercise a practical supervision" over all the agents of the Board, except the police. This Sanitary Superintendent is to receive a salary not exceeding five thousand dollars, and is to have two assistants at three thousand five hundred dollars each, one of whom must reside in Brooklyn, and perform the duties of Sanitary Superintendent for that city. He is to report in writing to the Board weekly, or oftener, on the work of his subordinates, and the sanitary condition of the district.

There are, further, to be appointed by the Board, fifteen district inspectors, ten of whom must be skilful physicians, acquainted with the districts in which they serve; and each of these is to report in writing twice each week, "stating what duties he has performed, and where he has performed them, and also such facts as have come to his knowledge connected with the purposes of this act, as are deemed worthy the attention of said Board, or as its regulations may require of him."

The amount of authority vested in the Board is immense; absorbing and exercising as it does exclusively all the powers and duties in regard to the sanitary management of this city and Brooklyn, which have heretofore been exercised by various individuals and bodies under a number of laws and ordinances. Peremptory power is given it to remove nuisances of every description and kind; to cause the cleansing and purification of alleys, streets, sewers, tenement-houses, and water-craft, and to take such other steps as may be necessary to insure the safety from disease of those persons residing in such vicinities.

In cases of impending pestilence, or in the event of the presence of a pestilence, it shall be the duty of said Board "to take such measures, to do and order, and cause to be done, such acts, and to make such expenditures (beyond those duly estimated for or provided) for the preservation of the public health, as it may in good faith declare the public safety and health to demand, and the Governor of the State shall also, in writing, approve. But the exercise of this extraordinary power shall also, so far as it involves such excessive expenditures, require the written assent of at least six members of the Board."

The Board is to keep a register of births and deaths; to inspect weights and measures; to co-operate with the Quarantine Commissioners; to communicate important sanitary information to the health authorities of other parts of the State; to charge no fees; to enforce the laws relating to the sale of unwholesome food and deleterious drugs; to attend to vaccination, and other preventive sanitary measures; to report annually to the Governor of the State the sanitary condition of the district, together with the laws and regulations of the Board, and its expenditures; and on or before the 10th of May in each year, to publish a "Code of Health Ordinances," violations of which are to be punished by the courts by fine.

A very important and useful feature is, the keeping open of a public complaint-book, in which citizens may notify the Commissioners of any nuisances which require to be abated. By this provision the Board will be enabled in a very direct manner to take prompt cognisance of any minor matters which might, perhaps, escape the vigilance of the subordinate officers. A sanitary engineer is to be employed, who shall survey and examine all premises and localities, and make such reports to the Board as it may order. Ample provisions are made for the employment of the necessary number of clerks and subordinates.

Since the passage of this act, on the 19th of February, the Sanitary Commissioners have been appointed by the Governor, and confirmed by the Senate. The gentlemen composing the Board have been well chosen, and, individually and collectively, are such as have the entire confidence of the profession and the community. They have already organized, have had several meetings, and have fairly initiated the great work which is before them.

Our profession is well represented in the Board by Drs. W. Parker, J. O. Stone, James Crane, of Brooklyn, the Sanitary Commissioners; Dr. John Swinburne, the Health Officer, Dr. E. Harris, the Registrar of Records, and Dr. E. B. Dalton, the Sanitary Superintendent. All of these gentlemen are acknowledged to be well qualified for their respective places. With the ample provisions of the bill, and the efficient aid which they will receive from the Police Department, and from the other members composing the Board, we have a right to expect such an enforcement of sanitary regulations as will tend to make New York a model city for cleanliness, and one of the healthiest upon the globe.—*N. Y. Medical Record*, March 15, 1866.

